# Challenges and opportunities in rapid disaster research: lessons from the field in New Mexico and Vanuatu

**DOI:** 10.3389/fsoc.2023.983972

**Published:** 2023-04-20

**Authors:** Mary Alice Scott, Kathryn M. Olszowy, Kelsey N. Dancause, Amanda Roome, Chim Chan, Hailey K. Taylor, Andrea Marañon-Laguna, Emilee Montoya, Alysa Garcia, Claudia Mares, Beverlyn Tosiro, Len Tarivonda

**Affiliations:** ^1^Department of Anthropology, New Mexico State University, Las Cruces, NM, United States; ^2^Department of Anthropology, Binghamton University, Binghamton, NY, United States; ^3^Department of Criminology, Anthropology, and Sociology, Cleveland State University, Cleveland, OH, United States; ^4^Département des sciences de l'activité physique, Université du Québec à Montréal, Montreal, QC, Canada; ^5^Bassett Research Institute, Basset Healthcare Network, New York, NY, United States; ^6^Department of Parasitology and Virology, Osaka Metropolitan University, Osaka, Japan; ^7^Department of Health and Human Services, Las Cruces, NM, United States; ^8^Ministry of Health, Port Vila, Vanuatu

**Keywords:** rapid research, COVID-19, natural disasters, rapid ethnographic assessment, U.S.-Mexico border region, Vanuatu

## Abstract

Rapid research is essential to assess impacts in communities affected by disasters, particularly those communities made “hard-to-reach” due to their active marginalization across history and in contemporary practices. In this article, we describe two rapid research projects developed to assess needs for and experiences of communities hard-hit by disasters. The first is a project on the COVID-19 pandemic in southern New Mexico (USA) that was developed to provide information to local agencies that are deploying programs to rebuild and revitalize marginalized communities. The second is a project on population displacement due to a volcanic eruption in Vanuatu, a lower-middle income country in the South Pacific, with mental and physical health outcomes data shared with the Vanuatu Ministry of Health. We describe the similar and unique challenges that arose doing rapid research in these two different contexts, the potential broader impacts of the research, and a synthesis of lessons learned. We discuss the challenges of rapidly changing rules and regulations, lack of baseline data, lack of survey instruments validated for specific populations and in local languages, limited availability of community partners, finding funding for rapid deployment of projects, rapidly training and working with research assistants, health and safety concerns of researchers and participants, and communicating with local and international partners. We also specifically discuss how we addressed our own personal challenges while also conducting time-intensive rapid research. In both studies, researchers shared results with governmental and non-governmental partners who may use the data to inform the design of their own relief programs. While different in context, type of disaster, and research strategy, our discussion of these projects provides insights into common lessons learned for working with communities at elevated risk for the worst outcomes during disasters, such as the need for flexibility, compromise, and good working relationships with community partners.

## 1. Introduction

Disasters necessitate the development of rapid research to assess constantly changing on-the-ground situations, particularly for communities made “hard-to-reach” through historical and contemporary practices of marginalization. In this article, we describe two rapid research projects developed to assess needs, experiences, and physical and mental health outcomes for communities hard-hit by disasters. The first is a project on the COVID-19 pandemic in southern New Mexico (USA) that was deployed, in part, to provide information to local agencies that are developing programs to rebuild and revitalize marginalized communities. This project consisted of a mixed-methods approach utilizing surveys, interviews, and collection of biological samples (dried blood spots) to explore potential connections among food insecurity, psychological distress, and management of type 2 diabetes. It utilized a research team of undergraduate students, graduate students, and faculty and included quantitative and qualitative data collection components. The first study phase focused on rural community members' general experiences with COVID-19 and related public health orders and restrictions. It included a one-time survey and an optional qualitative interview that further explored participant experiences. The second study phase specifically focused on the experiences of rural community members who had been diagnosed with type 1 or type 2 diabetes prior to the pandemic. This phase included another one-time survey focused specifically on issues related to managing diabetes during COVID-19 and a series of interviews to assess change in participants' experiences and perspectives as the conditions and restrictions of the pandemic itself changed.

The second project focused on population displacement due to a volcanic eruption in 2017 in Vanuatu, a lower- middle income country in the South Pacific, with mental and physical health outcomes data shared with the Vanuatu Ministry of Health. This project consisted of collecting survey data, anthropometric measurements, and biological samples (dried blood spots and hair). The research team included faculty from multiple institutions from the US and Canada, and close collaboration with local Ministry of Health officials and nurses. The survey, measurements, and samples were collected in a single month-long field session by the research team. The survey assessed participant experiences with housing, food, and water insecurity, their perceptions of the government and NGO (non-governmental organization) response, and psychological distress associated with displacement following the disaster. Anthropometric measurements and biological samples assessed physical health outcomes including blood pressure, physiological stress, and inflammation.

The results of these studies have been published elsewhere (Zahlawi et al., 2019; Olszowy et al., 2022). In this article, we describe the similar and unique challenges that arose doing rapid research in these two different contexts using different research designs, the potential broader impacts of the research, and a synthesis of lessons learned. We discuss the challenges of rapidly changing rules and regulations in the context of the ongoing pandemic and displacement, lack of baseline data, lack of survey instruments validated for specific populations, limited availability of community partners who were focused on addressing immediate disaster-related issues, and challenges in navigating the bureaucracy of university financial systems. Many of these issues are common for teams conducting rapid research and have been noted in the literature on disaster research generally, as well as in the course of research during COVID-19 specifically (e.g., Vindrola-Padros et al., 2020; Richardson et al., 2021).

We also discuss how we addressed our own personal challenges related to conducting time-intensive rapid research in a disaster setting. Discussion of personal challenges in research was uncommon in the literature until the COVID-19 pandemic (Moloney et al., 2020; Luciani et al., 2021), although these issues certainly have long been present for researchers conducting work in disaster contexts. Personal challenges during our COVID-19 project included the stresses of risk of transmission during a pandemic; the need to balance personal impacts of the pandemic, including serious illness of family members, with the day-to-day research tasks; adjusting to social isolation; and the psychological impact of witnessing the consequences of the disaster. Attending to personal safety was also a major challenge during our work in Vanuatu, as well as meeting basic needs (food, water, and shelter) during an ongoing environmental hazard without burdening the local community. Finally, the Vanuatu research team faced challenges in dealing with psychological distress associated with witnessing the impacts of the displacement on the community.

In our view, the benefits of conducting these research studies outweigh the challenges. In the context of our New Mexico study, we have presented our data to governmental and non-governmental organizations who are using it in their design of COVID-19 relief and health equity programs, and we have been able to train students at the undergraduate and graduate level to participate effectively in rapid research—a skill that will benefit them and the communities in which they work in the future. We have also conducted focus groups to present results of our research back to the communities in which we collected it, something not always possible in the context of disasters, and only made possible here when the local community centers re-opened as the pandemic declined. Proliferation of remote communication technologies such as Zoom also assisted ongoing communication with local health agencies regarding the project during the pandemic. Research in Vanuatu resulted in reporting data collected to local health authorities, and the results enabled authors Dancause, Olszowy, Roome, and Chan to apply for funding for a follow-up project examining longer-term impacts of the displacement. This highlights the need for immediate baseline data collection at the beginning of the disaster to later contextualize the long-term impacts. We were also able to provide a simple non-communicable disease (NCD) risk screening, giving participants information as to whether they needed to follow-up with the local health post. Finally, we also provided an evaluation of how psychosocial support received, including from the Ministry of Health worker interventions, influenced psychological distress.

While the projects are different in context, type of disaster, and research strategy, our discussion provides insights into common lessons learned for working in communities at elevated risk for the worst outcomes during disasters, such as the need for flexibility, compromise, and most importantly, good working relationships with community partners.

## 2. Rapid disaster research in “hard-to-reach” communities

Rapid research is deployed when data must be collected systematically and quickly, such as when information is needed to answer an immediate question or launch an emergency intervention, and/or when data are perishable or prone to retrospective bias. While rapid research has many applications, including in health care, marketing, and product development, we focus here on rapid research conducted in the context of human-centered experiences and impacts of “natural” disasters. Disasters may be precipitated by natural hazards, such as storms, volcanoes, or infectious disease, and/or may be due to human-made crises, such as conflict, war, and industrial accidents. Some authors (O'Keefe et al., 1976; Smith, 2006; Puttick et al., 2018) argue that no disasters are “natural”, given that what makes something a “disaster” is the lack of adaptive capacity within a community or population to adequately weather the consequences of the event. All types of disasters have significant effects on physical and mental health among the affected population, and in the case of those precipitated by natural hazards, these may include feelings of grief, loss, and uncertainty, post-traumatic stress, difficulty in accessing care and treatment for acute and chronic conditions, exposure to environmental stressors like infectious agents and toxins, dramatic changes in lifestyle, as well as increased food, water, energy, and housing insecurity. Rapid research in these contexts can assess the immediate and long-term mental, physical, and social needs and status of the community, provide information as to where limited aid resources may be best targeted, provide information for development and evaluation of targeted mental and physical health interventions, as well as contribute to improving local capacity in the face of future events.

We also focus here on rapid disaster research in so-called “hard-to-reach” communities. This label is often a black box that obscures the process by which communities become “hard-to-reach”. Communities are not inherently hard-to-reach. Instead, they are made so by structural forces such as institutional and interpersonal racism and sexism, other forms of discrimination, colonialism, geographic and/or social isolation, legal status, and belonging to a stigmatized group (e.g., HIV, substance use). “Hard-to-reach” communities are often also vulnerable and marginalized from access to resources and power. For example, many of the rural communities in southern New Mexico where our COVID-19 studies took place are classified as “colonias” because they lack a municipal government structure that provides basic services such as road repair and broadband access. The quality of roads can make the community physically hard-to-reach, while limited access to the internet can make it challenging to get information to communities when face-to-face contact is limited. In addition, southern New Mexico communities have experienced extractive research in which researchers come to communities, collect data, and do not return to report results or communicate the impact of the research. Community members are understandably wary of any future proposals to conduct research and may choose not to participate.

Our example study in Vanuatu extends the definition of “hard-to-reach” communities outside of the Western industrialized context where the term is usually applied. While Vanuatu is geographically hard-to-reach for some international researchers due to its location in the South Pacific, this is not why we apply this category here. Rather, factors including infrastructure and colonial history impact engagement of the local population with disaster aid as well as international research partners. For example, absence of paved roads on many islands and tenuous connection to urban areas via boats and small airports make distribution of aid during disasters difficult, much less travel for fieldwork purposes. Communication is also difficult due to uneven and limited access to the internet; the Vanuatu National Statistic Office reported in 2016 that only 20% of households had internet access, although over 80% had access to mobile phones. The former makes communication with international partners difficult while overseas, but the latter can facilitate communication with partners and research participants while in the field. Vanuatu's colonial history as a British-French condominium directly impacts the type of engagement officials and local residents seek with international partners. Vanuatu gained its independence in 1980, and in 1985 the government blocked foreign research as part of its effort to officially end colonial practices. Lasting 10 years, Taylor and Thieberger (2011, p. xxvii-xxviii) credit this moratorium with the establishment of an official focus on collaborative research in all sectors. This has not completely stopped a pattern of “parachute science”, whereby researchers from high income countries gather data *via* fieldwork and then return home without acknowledging local contributions or reporting data back to the communities. The international members of the Vanuatu research team have at times experienced some (proper and valid) questioning of our motives by local residents and officials given this history, and thus have always prioritized seeking the Ministry of Health's engagement in research design and deployment, providing credit for Ni-Vanuatu partners in publications and grants, and reporting all results back to both the Ministry and communities where the studies took place.

## 3. Special considerations in “hard-to-reach” communities

Rapid research within “hard-to-reach” communities in the context of disasters is essential because, due to pre-existing social and structural conditions, these groups may be the hardest hit in the immediate aftermath of the disaster, and most prone to long-term disruptions in access to resources including, but not limited to, food, water, shelter, energy, health care, and services and interventions designed to help communities recover. Because of their unique qualities, researchers planning to work with these kinds of communities must make special considerations regarding how to implement an ethical, feasible, and effective study. There is an extensive literature on conducting rapid disaster research, so we highlight here a few issues of special consideration when working among “hard-to-reach” groups.

First, individuals within “hard-to-reach” communities may also share certain conditions that define them as a “vulnerable” group in human subjects research, such as belonging to a minority social group or due to economic disenfranchisement. The US National Bioethics Advisory Commission defined individuals as “vulnerable” in research “either because they have difficulty providing voluntary, informed consent arising from limitations in decision making capacity (as in the case of children) or situational circumstances (as in the case of prisoners), or because they are especially at risk for exploitation (as in the case of persons who belong to undervalued groups in our society)” (NBAC, 2001, p. 85). Working with these groups even under “normal” circumstances requires consideration of additional safeguards to ensure consent is not coerced and risks are minimized (Gordon, 2020).

Second, in the context of rapid disaster research among these groups, a major question is whether disasters amplify or introduce vulnerability by reducing decision-making capacity through effects on psychological health. There is some disagreement in the literature on this point, and disaster-exposed individuals are not currently considered a special vulnerable category under US federal regulations for human subjects research. Collogan et al. argue that “disaster-affected populations should not necessarily be considered ‘vulnerable”' in the regulatory sense, but that they may be more vulnerable in the colloquial sense of “sometimes requiring additional care and attention” (Collogan et al., 2004, p. 369) due to psychological harm and inability to access necessary resources. However, Ferreira et al. (2015) suggest that psychological distress can indeed increase vulnerability in the regulatory sense, given that conditions like post-traumatic stress may alter decision-making capacity. The authors recommend that participants be screened for mental impairments that may alter their ability to make an informed decision about participation before research commences. Along these lines, the Substance Abuse and Mental Health Services Administration's Disaster Technical Assistance Center recommends that researchers “regularly consult with a mental health practitioner or be trained on how to address emotional distress” (SAMHSA, 2016). We additionally recommend that researchers draw from the practice of trauma- informed health care to inform research practices, particularly consent, with communities made hard-to-reach. These practices include creating safe environments for participants, recognizing signs and symptoms of trauma, and avoiding re-traumatization when possible (Menschner and Maul, 2016). This discussion raises an additional point that if a mental health condition is identified, the research team should provide resources for psychological support if available. This requires that researchers be familiar with on-the-ground availability of professional and/or traditional support networks (e.g., community leaders, religious leaders, etc.). We recommend Collogan et al. (2004); Ferreira et al. (2015), and SAMHSA (2016) for further reading on these and additional ethical considerations for research among vulnerable and/or disaster-affected populations.

A third important consideration when planning rapid research in communities made hard-to-reach is recruitment and retention. These groups are also frequently underrepresented in research in general, and funders like the National Institutes of Health have begun requiring plans to enroll diverse communities (e.g., gender, racial, and ethnic minorities) in research proposals in order to address inequities (Langer et al., 2021). As noted earlier, there are a wide variety of conditions that make a group “hard-to-reach”, and the reasons why a particular group may be difficult to enroll in research should be carefully considered. Existing literature suggests several strategies that may be effective across populations. For example, Bonevski et al. (2014) highlight involving community partners to improve subject sampling, as well as to act as “cultural brokers” to help improve relationships between potential participants and the research team. The authors also note that rapid research among these groups may at times need to rely on non-probability convenience sampling strategies due to time and resource limitations, such as “snowball sampling”, where enrolled individuals recruit new participants. Langer et al. (2021) also highlighted community partnerships as an important first step in developing and implementing research among underrepresented and vulnerable groups, as well as other strategies such as hiring study staff from the target community and approaching potential participants “where they are”. This refers not only to finding participants where they are physically located, such as at community centers or health fairs, but also where they are in terms of readiness to participate (Langer et al., 2021). This latter aspect is especially important to consider during a disaster to time recruitment of potential research participants to *after* their basic needs have been met.

Finally, collaboration with community partners is essential in rapid disaster research for many additional reasons beyond subject recruitment. For example, in the context of environmental health research following the 2010 Deepwater Horizon Oil Spill, Lichtveld et al. (2016) highlighted advantages of community-academic partnerships in disaster research that are applicable to rapid research in “hard-to-reach” populations, including “...assuring research projects target practical and relevant research questions and innovative answers, to improving environmental health risk assessment, management and communication practices by generating locally relevant data, implementing community-driven interventions, and disseminating culturally-tailored information” (Lichtveld et al., 2016, p. 3). We additionally argue that inclusion of community partners is ethically necessary to avoid or ameliorate undue burden on the community during the disaster and its aftermath, as well as mitigating potentially extractive or “parachute” research practices.

## 4. Rapid research data collection methods

Disasters necessitate rapid research due to the often-unanticipated nature of their occurrence, quickly changing on-the-ground circumstances, the potential loss of data due to destruction of records or inadequate resources to collect new information, as well as potential bias in collecting retrospective accounts from participants. In quickly developing situations, when the speed at which data is collected matters both for the ability to conduct research at all and for the likelihood that the research will have a positive impact on the situation, traditional anthropological methods may be inappropriate. However, the holistic perspective of anthropology still offers critical framing for rapid research in that it may incorporate both qualitative and quantitative methods, as well as approach an issue from more than one angle. These kinds of approaches are especially important when studying health-related outcomes. A number of anthropologists have developed rapid assessment methods to address these kinds of situations, and while quantitative assessment may be the most “rapid” of rapid approaches, it is not the only kind of data that is needed in disaster situations. And, as Vindrola-Padros et al. (2020) point out, rapid research is not the same as rushed research. Carefully planned and executed research processes, adapted for use during disasters, is still possible. Methods discussed in this section include surveys (some developed for the purpose of evaluating disasters and some repurposed), collection of biophysical measurements and samples to measure specific biomarkers, and rapid ethnographic assessments (REAs).

### 4.1. Quantitative surveys

Surveys are probably the most common method used to collect data on community, organization, household, and individual experiences and outcomes associated with disasters, and depending on the research question, there are numerous examples of published instruments available. Surveys are useful in that they are convenient for participants and researchers because they can be completed quickly, can be relatively inexpensive to deploy, and can cover a broad range of topics in a short amount of time. Surveys also have drawbacks, including that it may be difficult to infer complex interactions/connections (especially in terms of surveys given at one time point), are prone to respondent bias (e.g., memory recall), and may also be difficult to deploy successfully across different cultural/linguistic contexts if appropriate cross-cultural adaptation of the instrument has not already been conducted. “Cross-cultural adaptation” of instruments refers to not only linguistic translation, but also to cultural translation meant to ensure that specific constructs retain meaning across contexts (Beaton et al., 2000). The importance of cross-cultural adaptation of survey instruments has been well described elsewhere, along with various strategies and pitfalls in the process of adaptation; for examples, see Beaton et al. (2000) and Epstein et al. (2015). Given the nature of “hard-to-reach” groups, it is less likely that culturally adapted instruments already exist, and it is likely not possible to adequately adapt a survey in time for deployment in the case of rapid research. This is where surveys collected as part of REA (discussed below) may access richer contextual information to help place survey data within a broader sociocultural context, and elucidate meanings of culturally-specific constructs as well as connections among constructs and experiences. Biocultural anthropologists have written on the utility of ethnography to inform this kind of quantitative work more generally (i.e., beyond adapting existing surveys), and particularly on strategies for defining and operationalizing key cultural variables. We recommend Dufour (2006) as an introduction to this approach.

Our work in both New Mexico and Vanuatu has primarily been concerned with individual experiences and perceptions, as well as physical and mental health outcomes among individuals from specific communities. We have used both published instruments as well as developed-for-purpose questionnaires in both projects described in this article. As it is not the object of this work to catalog all instruments available, we highlight a few here. Both of these projects assessed psychological distress, which refers to the experience of mental and emotional suffering, including symptoms that may be shared with depression and anxiety. We did not measure specific mental disorders, as psychological distress aims to measure these feelings in association with specific events or current conditions that affect psychosocial responses. In our study in New Mexico, we used the Kessler 6-item Psychological Distress Scale to assess general psychological distress. This tool has been used in many other studies during disasters, including the COVID-19 pandemic, as well as during natural hazard-induced population displacement. Also during our COVID-19 study, we were interested in knowledge and behaviors associated with the pandemic and the local response. We used sources such as the World Health Organization's “Survey tool and guidance: Rapid, simple, flexible behavioral insights on COVID-19” handbook to design that portion of our survey (World Health Organization, 2020). In Vanuatu, we used the Impact of Events Scale-Revised (Creamer and Bell, 2003), which was developed to assess perceived distress associated with traumatic events. This scale has been used to assess distress across multiple studies, from those associated with Hurricane Katrina (e.g., Paxson et al., 2012) to more currently during the COVID-19 pandemic (e.g., Tee et al., 2020). Our surveys developed for both research sites also used questions developed in consultation with community-based experts, as well as applying our own ethnographic knowledge of the local context based on our long experience working in these communities.

### 4.2. Biophysical measurements and samples

Anthropologists who study physical and mental health-related questions in field settings are often tasked with collecting biophysical data under less-than-ideal conditions; these conditions may include geographical remoteness of the field site, absence of secure storage and refrigeration, inadequate or absent laboratory facilities or lack of trained technicians for point-of-care testing, as well as lack of resources such as stable power and potable water. In order to meet these challenges, biological anthropologists are at the forefront of developing and field-testing methods for biomarker data collection that are more cost effective, more easily transportable, easier to store, less prone to degradation, and importantly, less invasive for the study participants. These qualities also make these methods useful in rapid disaster research settings, which may share characteristics with remote field sites given potential disruptions in transportation, infrastructure, and municipal services. There are many field-friendly data collection methods that can be effectively used to rapidly collect data during a disaster. Anthropometry is a useful tool for quickly and systematically assessing nutritional status and can require little more than a scale, measuring tapes, and skinfold calipers, depending on the outcome of interest. Dried blood spots (DBS) have an increasing number of applications in field settings where collection, transportation, and storage of blood/plasma samples may be difficult; we recommend McDade et al. (2007) and McDade (2014) for a primer on benefits and drawbacks of DBS. Biomarkers that may be analyzed in DBS include metabolic, endocrinologic, and immunologic indicators, as well as antibodies to specific infections. Importantly, Ostler et al. (2014) also note that the ease and noninvasive nature of DBS collection means that research subjects may be more likely to participate in data collection. Temporally, DBS may be most useful for addressing questions about a more contemporaneous period (e.g., past days or weeks), while other materials, such as hair, can provide retrospective information about health, stress, and nutrition over the previous months (given that hair grows at approximately 1 cm per month) (Harkey, 1993). Other non-invasive specimens to consider, depending on resources (e.g., availability of freezers) and research questions, include urine and saliva (Ostler et al., 2014), which can also be dried for some analyses. Researchers should carefully review published collection protocols as analytes of interest may degrade in biological samples at different rates over time.

An additional advantage of DBS, hair, urine, and saliva to note in the context of disasters where in-person data collection is difficult (e.g., pandemics) is that subjects can be instructed on how to collect these materials themselves, making remote collection through mail a possibility (indeed, multiple for-profit companies already capitalize on widespread public interest in home-health testing products). However, we note that not all populations are equally accessible using these methods. During the New Mexico study, we were cognizant that not all individuals in the county were equally accessible by mail; not all people living in the county have a home address or PO box, for example. Remote collection would also not be feasible in Vanuatu, except in the case of a longitudinal study where participants could be instructed on collecting samples and how to store them until the return of the researcher.

In our studies described here, we used biomarkers to assess blood glucose management among people diagnosed with diabetes (New Mexico) and chronic disease risk and physiological stress (Vanuatu). In New Mexico, we conducted data collection remotely as well as in person. We contracted ZRT Laboratory to analyze hemoglobin A1C in DBS collected either by a research assistant in-person, or by the participant in their home. A1C provides a retrospective measurement of average blood sugar over the previous 3 months, and is a widely used indicator of diabetes management (The A1C Test and Diabetes, n.d.). Participants who completed the test at home were mailed a collection kit with instructions, and then returned their samples to the researchers in pre-paid envelopes. Samples were frozen in the NMSU Biological Anthropology Laboratory before being sent to ZRT Laboratory in batches. This allowed both the participants and the research team significant flexibility in collection and analysis over several months of field work under changing conditions (i.e., transition from remote-only data collection to carefully conducted in-person field work).

In Vanuatu we sampled hair to assess chronic physiological stress by assaying cortisol, the primary stress hormone produced by the hypothalamic-pituitary-adrenal axis (Chu et al., 2022), and DBS to assay C-reactive protein, a nonspecific indicator of inflammation that may be elevated in response to chronic psychosocial stress (Johnson et al., 2013). Together these provide a long- and short-term view of physiological stress, and CRP may additionally provide insight into a potential pathway that links physiological stress to inflammation and eventual development of cardiometabolic dysfunction (Wilson et al., 2006), risk for which was assessed using anthropometric and blood pressure measurements. The cortisol in hair is stable at ambient temperatures, and the DBS were dried over 24 h, and then were frozen within 2 weeks of collection. The characteristics of these methods were important for our ability to collect biomarkers on a limited budget in a remote location. For a further discussion of field-friendly methods to assess psychosocial stress, broadly conceived, see Brewis et al. (2021).

The collection of biomarkers requires trust between the research team and the participants. This may be difficult in “hard-to-reach” populations given problematic encounters with the biomedical system and/or with researchers who conduct “parachute science”. In New Mexico, we encountered very few individuals who were not willing to provide the DBS sample, although unsurprisingly the number was greater among the remote participants. Participants appreciated receiving the tests, especially those who had been unable to visit a provider in-person for a point-of-care or laboratory blood test to adequately track their A1C over the course of the pandemic. In Vanuatu, we have a long history of work in the community which facilitated trust in our procedures. Communities are also very aware of the risk of “NCDs” (noncommunicable chronic diseases) and participants desired to have their blood pressure, weight, and other indicators checked before making a longer trip to a health post or hospital. We were concerned about lack of interest in providing hair and blood samples as there may be some cultural reticence to provide these items due to use of bodily materials in the practice of sorcery in some parts of Melanesia (Rio, 2019). However, we have not encountered systematic resistance to collection of these materials. Some of this may be due to adoption of medical pluralism across some parts of Vanuatu, where individuals are very familiar with biomedical practices which they use in tandem with “traditional” healing (Elliott and Taylor, 2020). This highlights why ethnographic insight into the community involved in the study is essential.

In both studies, we were careful to provide participants with their data where possible. We included information on clinical “cutoffs” that designate high-risk groups and recommended that individuals visit a primary care provider if their numbers were above these cutoffs. We note that as anthropologists, we are not medical providers, but it is ethically necessary to provide information on where treatment is available for those with clinically abnormal values. In New Mexico, information was provided on local free or sliding scale-fee clinics and in Vanuatu, individuals were recommended to go to the nearest aid post or dispensary staffed by a nurse for follow-up.

### 4.3. Rapid ethnographic assessment (REA)

REA is a data collection and analysis tool designed to collect qualitative data in situations that require rapid response. The ethnographic component of this type of assessment allows for the collection of rich contextual and locally relevant data that highlights the complex social, political, and economic factors that contribute to the conditions seen on the ground. This strategy is best used when data is needed quickly to assess an evolving situation such as a global pandemic or environmental disaster and is particularly useful to quickly assess conditions for “hard-to-reach” and historically/structurally marginalized populations. It allows for immediate engagement of community members who can provide practical insight into both their conditions and into potential solutions. REA is particularly useful when we know little about a problem and/or when the problem is in the process of development (an emerging situation). The potential methods used in REA are wide ranging and may include interviews, focus groups, observation, mapping, and surveys among other data collection methods. In addition, REA usually incorporates participants across multiple stakeholder groups.

Recent REA and other similar rapid research models on COVID-related experiences highlight the ways in which rapid research must be flexible and adaptable to quickly changing circumstances, while also maintaining fidelity to appropriate research practices. Key ideas include a focus on capturing voices not commonly heard to ensure that the experiences of those most affected by COVID-19 are known and addressed (Callejas et al., 2020), more rapid data analysis cycles that strategically utilize the resources of the team rather than relying on one individual (Callejas et al., 2020; Moloney et al., 2020; Palinkas et al., 2020) and conducting multiple stages of data collection and analysis simultaneously (Freidus et al., 2020; Vindrola-Padros et al., 2020; Luciani et al., 2021), a strategic focus on key areas in need of intervention rather than a broad comprehensive analysis (Callejas et al., 2020; Richardson et al., 2021) particularly during rapid rollout of public health interventions (Collins et al., 2022), operationalizing theoretical models to aid in rapid data analysis (Palinkas et al., 2020; Collins et al., 2022), and utilizing existing networks and collaborations to quickly deploy research strategies (Moloney et al., 2020; Luciani et al., 2021; Richardson et al., 2021; Collins et al., 2022). Others note that it is critical to continue the collaborations built during crises such that community voices continue to be heard in efforts at recovery and mitigation of future crises (Simpson et al., 2021) as well as to be responsive to the changing circumstances of collaborators (Richardson et al., 2021). It is critical to report data quickly to community collaborators and policy makers so that they can act on this data in the moment (Freidus et al., 2020; Vindrola-Padros et al., 2020). This kind of regular and timely communication can help to build these collaborations for the future and facilitate the immediate use of data for policy change.

In the context of the studies discussed in this article, several key components of REA were implemented. In the New Mexico study, interviews were designed to be conducted via phone or in person with specific protocols outlined for each method. Multiple shorter interviews were developed to facilitate the potential additional energy required when conducting an interview over the phone when body language and other visual cues and aids are not available to contribute to the interview process. These shorter interviews were conducted in rapid succession due to the potential for loss to follow-up as phone numbers change or are disconnected frequently. All members of the team were trained to conduct interviews and were thus able to maintain these more rapid research cycles. For example, multiple team members were available to conduct interviews in Spanish. In addition, some team members completed interviews more quickly than others because of fewer challenges in contacting and scheduling interviews with their assigned participants. They were then able to support research team members who had more trouble with interview scheduling, thus expanding the potential times available for interviews. While interviews were not conducted in Vanuatu, similar strategies were employed to complete surveys. Surveys could be completed with a research assistant or could be completed by the participant on their own and then reviewed by a researcher. This allowed for flexibility in data collection and allowed surveys to be completed quickly.

Research was also planned to conduct data collection and analysis simultaneously, or near simultaneously, in the New Mexico and Vanuatu studies. While it is common to begin preliminary analysis of data prior to the end of formal data collection, the process overlapped more than is typical for the New Mexico study in particular. Preliminary data analysis began immediately as surveys began to come in and following interviews. Analysis of interviews was modified to directly analyze data from the interview audio rather than waiting for transcription to be completed. During this time, some team members were also working on transcriptions that could be utilized in a second phase of analysis. The preliminary results were reported to community partners early rather than waiting for a final analysis to be complete. In Vanuatu, preliminary data analysis was conducted toward the end of the month of fieldwork so that authors Roome and Chan could present key information to the Vanuatu Ministry of Health partners before the investigators departed the country.

Finally, it was critical to focus the research on a shared area of interest between the research team and community partners in both New Mexico and Vanuatu. Community partners were able to justify the work required of their staff to support the project because it also met their own strategic goals and efforts to rebuild after the height of the COVID-19 pandemic (New Mexico) and during the ongoing disaster in Vanuatu. These partnerships were essential for rapidly identifying multiple methods for participant recruitment, strategizing remote methods for data collection with marginalized communities, and in the New Mexico study, transitioning to safe in-person data collection at community centers when that was allowable.

REAs may be difficult to implement given the slow-moving nature of research-related infrastructure. For example, university research systems may or may not be prepared to facilitate rapid research as normal strategies for processing funding and obtaining ethical approvals are often set up for longer time frames. Systems and processes may be complex and require the input of several different institutional units (Richardson et al., 2021). Research teams may also need to be constructed and managed differently. Rapid research during disasters may require bringing new research team members, including student trainees, on board quickly and training them more rapidly than in non-disaster situations (Luciani et al., 2021). Significant time may also need to be spent on administration and coordination of larger or less experienced research teams (Vindrola-Padros et al., 2020). In addition, research teams identified the need to build support systems into research processes to address the individual stressors of research team members and ethical challenges of research related to the pandemic (Luciani et al., 2021)—for example instituting weekly briefing sessions that included these discussions (Moloney et al., 2020). The need to consider whether the team *should* do research at any point during a disaster has also arisen, as research teams need to consider the potential for harm or benefit to the populations affected as well as the researchers (Vindrola-Padros et al., 2020).

As will be discussed further below, rapid response from the respective research team's IRB was critical in moving the projects forward in time. In the New Mexico study the IRB was able to rapidly enact processes to facilitate quicker approval for COVID-19 related projects as well as to guide researchers in developing safe protocols for data collection. The Vanuatu study was facilitated by a pre-existing ethics protocol that was modified and approved rapidly for the 2017 study. Processing of funding was another matter, however. New strategies for processing funding for research were not significantly changed and did cause delays for the New Mexico project. Consistent communication with university offices responsible for managing and monitoring funding was essential to limit these delays.

Availability of technologies like Zoom facilitated both projects and was especially important during the New Mexico study for training new researchers. The New Mexico study team additionally collaborated to train new research team members, including having seasoned student research team members conduct some of the training for new research team members. The team also had regular research team meetings online at first and then hybrid when some restrictions on convening in person were lifted. The hybrid format allowed for adequate physical distancing while offering some in-person support for those research team members who felt particularly isolated at the time of the research. Similarly, online meetings were critical for the deployment of the Vanuatu project, given that researchers were spread across Vanuatu, Canada, the US, and Japan.

Our studies demonstrate some of the many challenges of conducting rapid research in disasters, in particular balancing the need to work quickly and flexibly with maintaining the rigor of the study as well as maintaining relationships and networks through which the research may happen. Our studies contribute to this ongoing discussion and development of best practices for rapid research in communities labeled as “hard-to-reach”. As noted above, working in these communities presents particular challenges, but also offers creative new ways of engaging communities during disasters.

## 5. Rapid research in domestic (United States) context: the colonias COVID-19 studies

**Figure 1:**
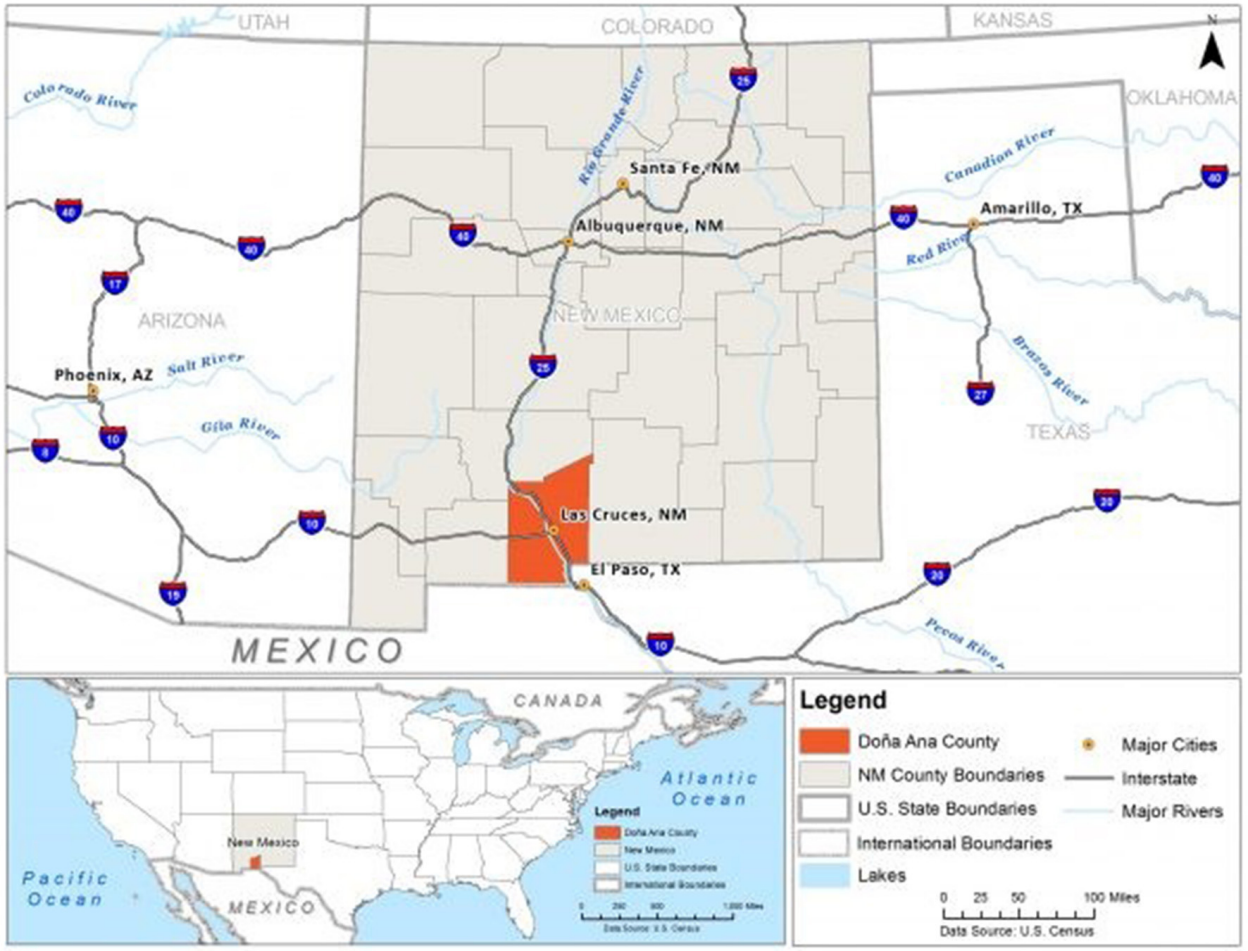
Map of Doña Ana County, New Mexico. Image Source: Subhashni and Raja (2018).

The U.S.-Mexico border region is typically defined as the geographic area 100 kilometers north and south of the international boundary. It includes 44 counties in four U.S. states and 80 municipalities in six Mexican states (U.S. Department of Health Human Services, 2017). Many areas within the border region are medically underserved, have high rates of poverty, and experience a range of health inequalities. Doña Ana County (DAC), New Mexico (Figure 1) is one of the U.S. counties within the border region. The 2021 population estimate is 221,508, 68.8% Hispanic/Latino, and 16.5% foreign born. Nearly half of the residents (49.4%) speak a language other than English at home, and 20.5% live in poverty (US Census Bureau, 2021). Doña Ana County contains 37 colonia communities. Colonias, in the U.S. context, are designated by the U.S. Department of Housing and Urban Development as rural communities, often unincorporated, located within 150 miles of the U.S.-Mexico border that may lack adequate infrastructure and services such as paved roads and sewer systems (Viva Doña Ana, 2022). DAC has identified several key issues within its colonia communities including unpaved roads that contribute to dust pollution and health problems related to pollution, and limited accessibility for first responders, health care workers, school buses and others, especially during flooding in the summer rainy season. The county's wastewater treatment plants serve approximately 10% of colonia communities (Doña Ana County, 2017). The average median household income in Doña Ana County's colonia communities was under $35,000 in 2010 and average household poverty levels neared 30% (American Community Survey, 5-year estimates 2006–2010). In addition, Doña Ana County is located between the U.S.-Mexico border and United States interior border checkpoints, which essentially traps some community members and families with mixed documentation status in the region. Author Scott has worked in DAC conducting research on health inequities and serving on community health organization advisory boards since 2013. The interests of one community health organization and those of authors Scott and Olszowy to better understand the impact of COVID-19 on the county's rural communities led to the project described in this article.

### 5.1. General methodologies

In response to the impact of the COVID-19 pandemic, authors Scott and Olszowy developed a research team including several New Mexico State University (NMSU) undergraduate and graduate students and a community health worker coordinator (author Mares) from the DAC Health and Human Services Department. The team designed a two-stage study to better understand the mental and physical health impacts of COVID-19 restrictions for rural communities. The first study included a survey and individual interviews and focused broadly on community members' experiences of food insecurity, mental health, and health care strategies. During phase 1 of the study, we noted specific patterns associated with people's ability to manage chronic health conditions and thus focused phase 2 of the study specifically on the experiences of people with a diagnosis of type 2 diabetes. This study also included both surveys and qualitative interviews as well as blood samples to estimate A1c. The long-term objective of these studies is to develop a model of how the COVID-19 pandemic contributes to outcomes in individuals with diabetes by exploring interactions among diabetes, psychological distress, and food insecurity.

### 5.2. Working with local collaborators

This project required the collaboration of the Doña Ana County Department of Health and Human Services (DAC DHHS) given the difficulty in recruiting individuals living in rural areas in southern New Mexico, which we discuss further below. Scott's previous connections to community health organizations facilitated a collaboration with DAC DHHS, which has active community centers in 12 rural communities across the county. We worked closely with DAC DHHS's community outreach coordinator to determine the best ways to reach community members for study recruitment, appropriate data collection strategies, and ways to report study results to study participants. The community outreach coordinator manages the teams working at community centers throughout the county and was able to connect our team to community health workers who supported the study by helping the team to engage with local communities.

### 5.3. Research specific challenges

#### 5.3.1. Ability to share information, recruit participants, and follow up

COVID-19 restrictions limited some of the usual means that our research team used to disseminate information about research and recruit research participants, particularly given that outreach to rural communities often requires face-to-face interaction at community events. Many individuals in Doña Ana County's rural areas do not have consistent access to internet, cell phones, computers, or other means to learn about research through electronic communication. Communication via mail is inhibited by the lack of street addresses and frequent change of address among rural community residents as well as phone service disconnection. Following initial recruitment of participants, follow-up was also more difficult given these same communication limitations.

#### 5.3.2. Changes to team communication and research team structure

Additionally, much of our communication within the research team had to be constructed differently to maintain research team safety during the height of the COVID-19 pandemic when the university required all research that could be conducted remotely to do so. Rather than in- person meetings, we conducted our research team meetings via Zoom until it was allowable to resume in-person meetings. Although we were able to utilize Zoom effectively, some discussions, particularly regarding qualitative data analysis, were more difficult as we could only share one screen at a time and could not easily review analysis completed using Nvivo, the qualitative data analysis software we used for the project. During the course of the project, research was permitted to move back to in-person methods so long as specific COVID-19 safety protocols were in place and approved by the NMSU IRB. While this was a benefit to the research, it was time intensive to design these safety protocols and ensure that they could be effectively implemented at community sites such as community centers. Ultimately, with the support of community partners, we were able to develop a hybrid format that maintained safety for both participants and research team members.

### 5.4. Personal challenges

#### 5.4.1. Changes to “normal” academic routines

Due to COVID-19, our research team members all had to shift away from normal routines for academic work as well as personal activities. For example, academic classes shifted to online and sometimes asynchronous formats. These adjustments to academic schedules were often disruptive for both students and faculty. In addition, working and studying at home often meant more frequent interruptions and less access to private space for team members.

#### 5.4.2. Changes to social interactions and increased isolation

Typical modes of social interaction were curtailed because public places and businesses were closed, which led to feelings of isolation for most on our research team. These changes created increased feelings of stress for the research team, who then saw similar responses reflected in the interviews and survey data for our participants as well. While mindfulness of the wellbeing of the research team is always a priority, during the pandemic we needed to increase our focus and time attending to research team wellbeing.

#### 5.4.3. Increased intensity of time online

As courses, meetings, and personal social time moved primarily online, research team members needed to take more frequent breaks from data analysis and other research-related activities. This need for additional breaks required a change in our typical timelines for research. Frequent adjustments to timelines were necessary throughout the course of the study.

#### 5.4.4. Heightened learning curve for students

Additionally, the students on our research team were new to conducting research. The learning curve is steep in non-pandemic times. Learning how to conduct research during a pandemic when normal research protocols for both mentors and trainees are impossible increases the learning curve even more.

#### 5.4.5. Fear of COVID-19

Across research team members, differing levels of fear of contracting COVID-19 led to challenges in structuring team processes. Some team members felt comfortable with some in person interaction, while others preferred to remain completely online. Team members also considered the vulnerability of our research participants in making decisions regarding in-person or continued online interactions. It should be noted that community members who participated in this research also had differing levels of fear of contracting COVID-19, and some preferred in person interactions much earlier in the research process than others.

### 5.5. Value of the work

The research team started this project because we saw the effects of the pandemic in our communities and wanted to use our research skills and/or learn how to conduct research to be able to address the effects of the pandemic. While some of our findings were expected—high levels of psychological distress, for example—others were not. Interview participants discussed unique strategies they used to manage and even improve their health during the pandemic. People mobilized social networks and used their newly found time to engage in new physical activity and/or healthier diets. The research team identified strengths and major challenges within rural communities that will be useful for local governmental agencies as they plan new programming to “rebuild” rural communities following the pandemic.

## 6. Rapid research in an international context: the Ambae displacement study

**Figure 2:**
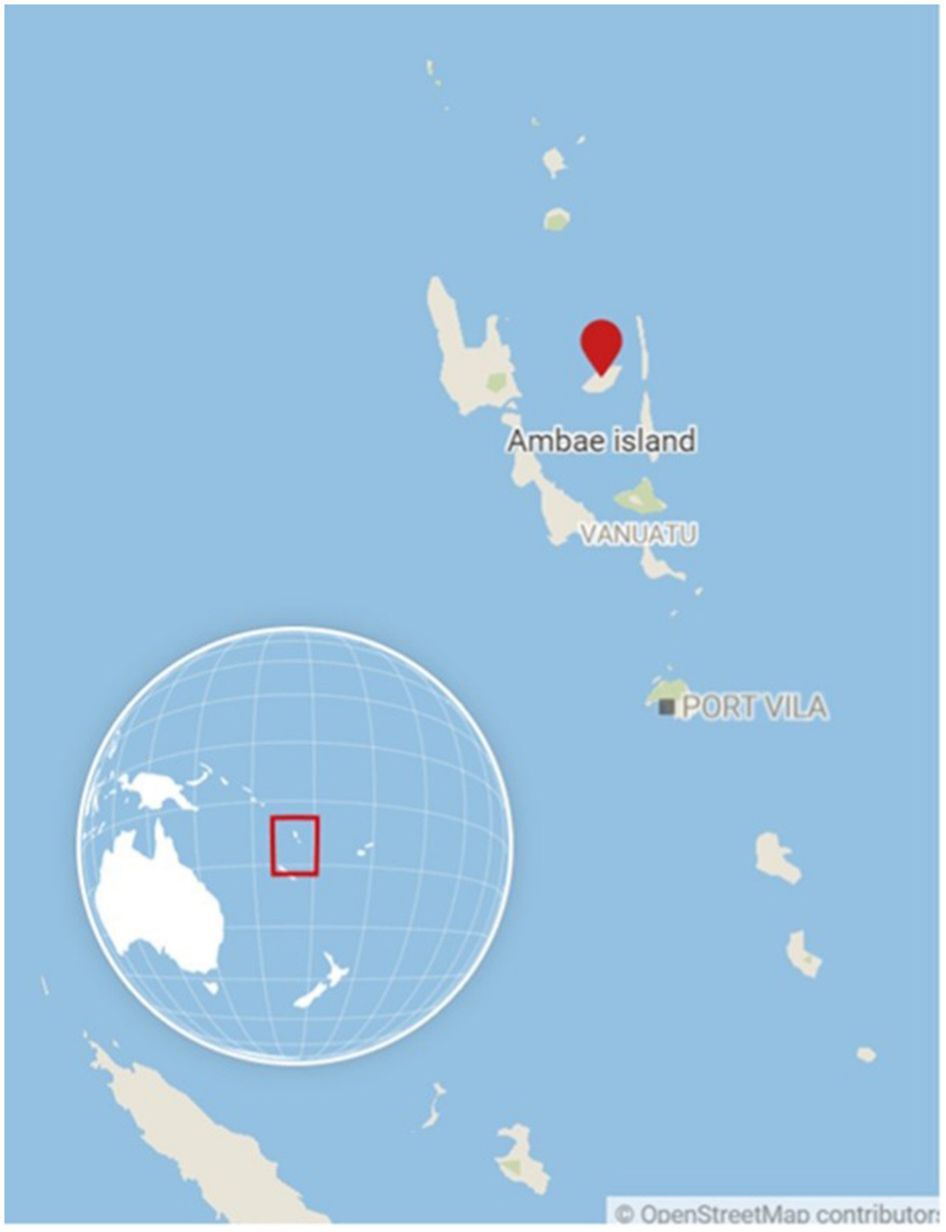
Map of Vanuatu with Ambae island indicated. Image Source: OpenStreetMap.

**Figure 3:**
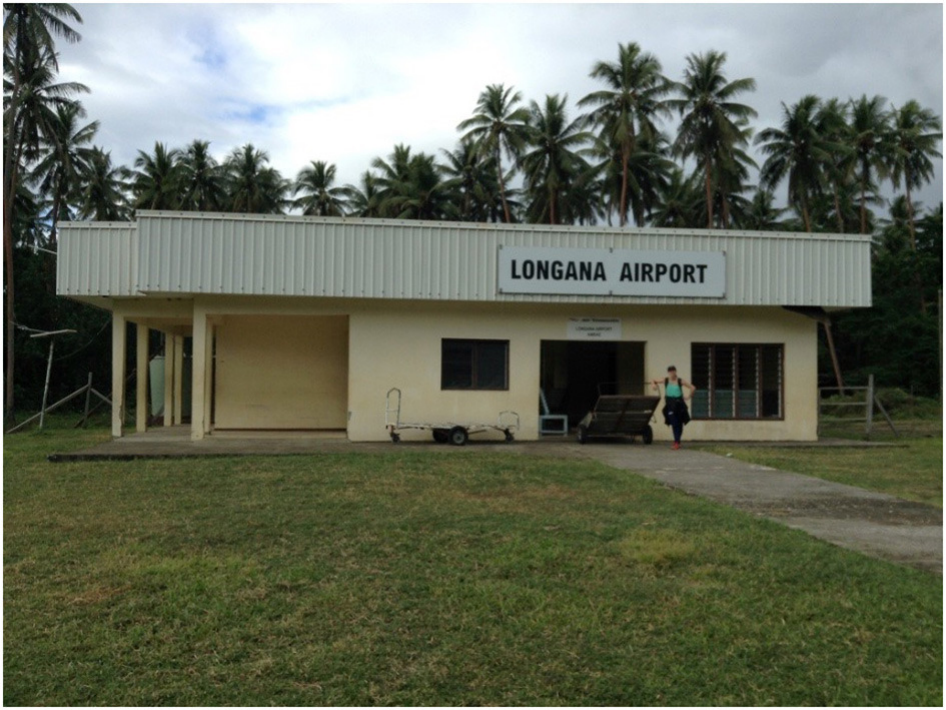
Longana Airport, located in Northwest Ambae. Image source: Kelsey Dancause.

**Figure 4:**
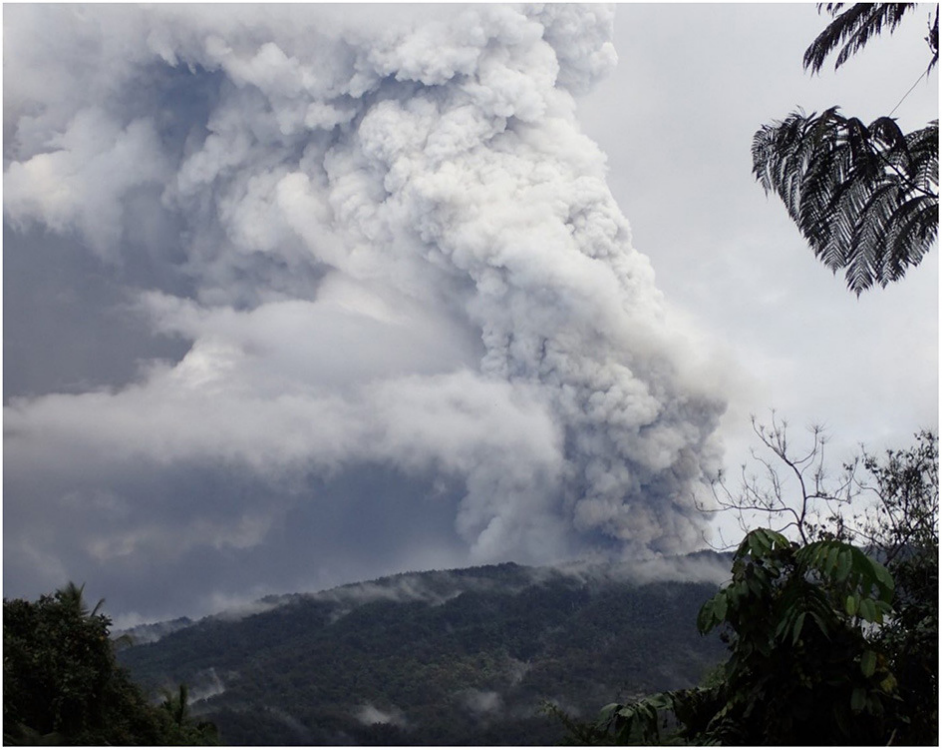
Eruption of Manaro Voui Volcano, Ambae island, Vanuatu in November/December 2017. Image source: Amanda Roome.

Vanuatu is a lower-middle income island nation in the South Pacific (Figure 2). The majority Melanesian population live across 63 inhabited islands within the Y-shaped archipelago, and most of the population (80%) engage in subsistence agriculture as their primary livelihood (UNSDG, n.d.). As of the 2016 Vanuatu National Statistics Office mini-census, approximately three-quarters of the 270,000+ population reside in rural areas like Ambae island. Ambae is located in Penama Province and has limited municipal infrastructure; for example, in 2009 over 80% of households identified pit latrines as their primary toilet facilities, 2% of households were serviced by private piped water, and fewer than 5% of households were on a serviced electric grid (Vanuatu National Statistics Office, 2009). Paved roads are also absent; the island must either be traversed via dirt roads/paths or by boat over the ocean. The island is connected to urban areas Port Vila on Efate island, Luganville on the island of Santo via flights departing from several small airfields (Figure 3) as well as by boat. Ambae (also called Aoba) (Figure 2) is an oceanic island formed by the Manaro volcano (elevation: 1,496 m). Manaro is the largest volcano in Vanuatu and was dormant until the 1990s when activity in 1995 and then 2005 led to short-term displacements of local villages to other parts of the island ['Aoba island', (n.d.)]. In October 2017, substantially elevated activity (Figure 4), including acid rain, ashfall, and flying rocks prompted an official evacuation order of the entire island population. In the end, 10,869 individuals were moved for a period of 4–6 weeks to nearby islands including Santo, Pentecost, Maewo, and Efate. Individuals underwent significant hardships including uncertainty about when and if they would be able to return home, destruction of homes and subsistence gardens, risks to health from exposure to ash inhalation and contaminated water, as well as food, water, and housing insecurity at emergency shelters.

Authors Olszowy, Dancause, Chan, and Roome have worked in Vanuatu for over a decade on questions related to economic development and health transitions (see: Dancause et al., 2010, 2011a,b, 2013; Olszowy et al., 2015, 2017, 2018; Sun et al., 2016; Weitz et al., 2017; van Horn et al., 2019), and have more recently begun to conduct assessments following natural disasters (see: Pomer et al., 2018, 2019; Zahlawi et al., 2019). We were personally and professionally interested in the impacts of the displacement on mental and physical health among the population given our previous research among the Ambae community. Population displacement is common and increasing due to factors including climate change and conflict (UNHCR, 2019, n.d.), and Pacific islands are at particular risk for disasters and hazards due to the former (Noy, 2015). More models are needed to explore factors that buffer and amplify effects of disaster-related displacement on individual and community health. The Ambae displacement was a particularly interesting model because (1) we have data on health-related behaviors and outcomes from this population dating to 2007; (2) the entire island population, rather than a subset, were displaced; and (3) the Vanuatu Ministry of Health deployed a first-of-its-kind mental health response, which provided an opportunity to evaluate the impact of professional intervention on psychological health during the disaster. Our previous work in Vanuatu, and unique resources within our research group, enabled our rapid response to the situation.

### 6.1. General methodologies

In response to the Ambae displacement and repatriation, we sent a research team (authors Chan and Roome) to the island to collect assessments of individual experiences and mental and physical health in the aftermath. Rapid response was crucial due to the retrospective nature of the study; we were concerned about the fidelity of participant memory of events as more time passed between the displacement and data collection. Additionally, most psychological health questionnaires are designed to assess the previous few weeks or months and are prone to recall bias over longer periods of time. We also collected anthropometric measurements and biological specimens (hair and blood spots) to assess factors including physiological stress (hair cortisol) and inflammation (C-reactive protein). These specimens also needed to be collected close to the event in order to assess the immediate impacts on physical health markers.

The initial displacement took place in October 2017, and data collection for this study took place in November-December 2017 after repatriation was underway. The research team traveled to 13 villages representing the four regions of Ambae (north, south, east, and west), and conducted the aforementioned procedures among adult men and women volunteers. The survey assessed participant experiences during displacement (e.g., food and water insecurity, perceptions of government and NGO response, receipt of psychosocial support), experience of psychological distress in response to the displacement, and physical health outcomes (e.g., blood pressure, physiological stress as measured by hair cortisol, and inflammation as measured by C-reactive protein in blood spots). Participants could choose to self-administer the survey, or have it read to them by a research assistant, and all measurements and sample collection were conducted by trained assistants. A local research assistant traveled ahead of the survey team to announce data collection at locations including clinics, churches, and community centers. Further description of study methods is available in Zahlawi et al. (2019).

The survey instrument used in this study was originally developed in response to a previous disaster in Vanuatu. In March 2015, the country was struck by Cyclone Pam, a category 5 cyclone that caused wide-spread destruction due to high-force winds, rain, and flooding. In all, 16 people were killed and a majority of Vanuatu's population required immediate aid (Coates, 2015). A research team directed by Dancause traveled to Vanuatu 3–4 months following the cyclone to assess the impact of the disaster on psychological health and nutrition among pregnant persons (Pomer et al., 2018, 2019). The survey included questions on experiences related to the cyclone, dietary diversity, and psychosocial distress. The latter questions were based on the Impact of Event Scale-Revised (IES-R) (Weiss and Marmar, 1997) (instrument is further described in Pomer et al., 2018, 2019). Having this instrument available for modification is a major reason why we were able to respond quickly to the Ambae displacement. Additionally, a human subjects protocol was already approved at investigator Dancause's institution, allowing for submission of a modification to the protocol which took ~1–2 weeks to review, rather than a new protocol which would have necessitated longer review.

Challenges in developing the survey were numerous. Mental health is a relatively new priority for the Vanuatu Ministry of Health, with no culturally-specific clinically-validated surveys available. Thus, we have had to rely on surveys developed in other contexts (typically in high-income, English- speaking populations). Translation was conducted by local research collaborators into Bislama, an official English-based pidgin language, which is spoken across Vanuatu. However, with over 100 different languages spoken across the archipelago, local variations of Bislama may affect the generalizability of surveys across communities. Despite these challenges, the distress scores derived from the Cyclone Pam survey did show indications of construct validation (i.e., the measure of distress was associated with factors that predict/are predicted by distress in other contexts). For example, distress was predicted by variables including dietary diversity and hardship (damage to village, home, and garden) (Pomer et al., 2019). Distress also was predictive of birthweight among babies born to persons who were pregnant during the cyclone (Pomer et al., 2018).

### 6.2. Working with local collaborators

Local collaborations have always been integral to our research in Vanuatu, and during the Ambae displacement study these collaborative relationships were key to our rapid response. The primary point of contact between the international research team and the Ministry of Health in Vanuatu is Director Len Tarivonda (author Tarivonda), who provides permission for research studies to be conducted on behalf of the Ministry, among other assistance. Once a meeting was established, Director Tarivonda was satisfied that the project focused on mental health issues as this fit with the current direction in public health focus at the Ministry. Director Tarivonda was also key in arranging for research assistance by professionals already working on Ambae, including Beverlyn (Bev) Tosiro (author Tosiro) and Maxley Malanga, who are local nurses, as well as Markleen Tagaro, the Penema Provincial Health Supervisor. Tosiro, Malanga, and Tagaro were instrumental in arranging all logistics on Ambae, including facilitating networking by speaking to local village chiefs and spreading the word around villages. They also held significant local knowledge, such as which villages were most affected, and when locally scheduled events were happening to help coordinate with the project objectives. These collaborators also had worked with some of the research team on other projects in the past, and so understood how to conduct outreach and data collection based on their experience during previous studies. In addition to Tosiro and Tagaro, the research team also hired local nurses or nurse aides who worked at dispensaries (medical aid posts) within the communities.

Response to our survey was overwhelming. Participants stated that they greatly appreciated being asked about their experiences. We also reported back to Director Tarivonda at the conclusion of the data collection period and were able to communicate not only information from our surveys, but also other ongoing hardships experienced by the community. For example, there was some concern among community members about the aid received from organizations like the Red Cross, Save the Children, and Australian Aid. Households received one bag of rice and one bag of water from these organizations, and while appreciated, the aid was not sustained; with gardens destroyed and water sources contaminated by ash, fear over lack of resources contributed to ongoing distress. Although our collaboration with the Ministry of Health is longstanding, we did experience several challenges. First, it was initially (but understandably) difficult to get in touch with Director Tarivonda due to the overwhelming impact of the disaster on his time and resources. We additionally faced some issues in communication with the local research assistants regarding the purpose of the project, and how payment for assistance would be distributed, due to a misunderstanding between the research team and our point-of-contact with potential research assistants in different villages. Given the short time period that we had for data collection, it was integral to the project that we work out these issues quickly and efficiently; our long-standing relationships with these collaborators and trust built with the local communities are largely what allowed us to work past miscommunications.

### 6.3. Research specific challenges

The rapid nature of this project created specific challenges not usually encountered in research that has been planned over months and years. These challenges included:

#### 6.3.1. Availability of colleagues for immediate travel

Several members of the research team are university faculty who were well into fall (northern hemisphere) semester courses and were thus not able to travel to the research site. We were fortunate that both Roome and Chan had extensive previous experience working in Vanuatu, and positions that allowed them to travel to the research site at short notice.

#### 6.3.2. Transporting field equipment to the field site

We encountered some difficulties in getting field equipment (e.g., filter cards for blood spots) to the appropriate person before departing for Vanuatu, and once traveling, also encountered issues regarding weight restrictions for air travel. We typically have a large team traveling to Vanuatu and are able to split equipment between multiple individuals, which was not possible in this case.

#### 6.3.3. Travel in a disaster-struck area

Ambae is a rural island and travel typically occurs by foot, boat, or in some cases, trucks on dirt roads. The research team was not only impacted by typical challenges in conducting research in this area (e.g., heavy tropical rain and subsequent mud making truck travel impossible; traveling south on the ocean to an atypical dock that made loading the boat difficult), but also significant personal danger due to the ongoing nature of the Manaro volcano eruption. Schedule changes regarding plane flights were also not communicated, likely due to disruptions related to the disaster, resulting in the research team missing a flight that departed 5 h earlier than scheduled.

#### 6.3.4. Funding for rapid research

Applying for funding for field research is typically a months or even years-long process. Very few resources are available for rapid research. The Natural Hazards Center in Boulder, Colorado provides funding for rapid disaster research, and we successfully applied to this resource to support international travel. The other major source of funding for this project was author Dancause's existing research funding from a provincial salary support program, which included some flexible funds to support her broader research program that could be used to support data collection.

### 6.4. Personal challenges

The field research team faced additional personal challenges while in the field. These included:

#### 6.4.1. Meeting basic needs

Due to the nature of the disaster, food and potable water was not widely available, and the research team did not want to exploit already-stressed local resources. Additionally, while water is readily available in rivers and streams, these were heavily contaminated by ash. The team thus had to pre-purchase and carry a large amount of food and water, in addition to their research equipment. Planning to meet basic needs while not burdening the host community is integral to rapid research in disaster scenarios.

#### 6.4.2. Maintaining physical health

Field work is physically demanding, and conditions were not improved by the disaster. The team put themselves at risk of ash inhalation as well as risks associated with contaminated foods. One member of the research team also acquired an *Escherichia coli* infection and was significantly ill during the latter part of the trip and travel home. Other risks included potential for malaria transmission (malaria is endemic on Ambae) as well as other infectious diseases (e.g., tuberculosis, hepatitis, and typhoid). The team acquired international travel insurance in case of accidents and death, and was also aware of “usual” risks due to previous research in the country, used filtered straws and bottled water as precautionary measures, and was in good physical fitness, but this does not preclude occurrence of unanticipated events.

#### 6.4.3. Attending to mental health

The researchers have worked in Vanuatu for over a decade and have many personal connections on Ambae. Witnessing any human suffering is distressing, and observing the hardships experienced by communities and individuals was detrimental to the team's mental health. In one particular instance, the team was struck by the severity of the disaster when they crossed from east to south Ambae, where the impacts of the eruption were greatest. Somewhat ironically, while our survey was assessing psychological health among Ambae residents, we neglected to fully consider the impacts on mental health among the research team. Field research is stressful even outside of disasters, and the preparation that our team had from previous work in Vanuatu did help buffer them from some of the effects.

### 6.5. Value of the work

Rapid research is challenging, despite the preparation that our team had due to many years of collaborative research in Vanuatu. It is thus important to consider the overall value of the research compared to the burden on the local community and government, as well as on the researchers. Our work on the Ambae displacement demonstrated both individual and community value. Ambae residents repeatedly expressed appreciation in being asked about their mental health, and an opportunity to discuss their experiences related to the disaster response. For example, many expressed concerns regarding the transient nature of outsiders (i.e., international aid organizations) coming to the island, distributing limited items to provide for basic/immediate needs, while not staying to work on neglected long-standing issues (such as the lack of infrastructure that made the disaster response more difficult). The government (Ministry of Health) also expressed gratitude for exploration of mental health during the disaster, given that this is a new national priority. Our instrument is an important first step in developing tools for assessing mental health at the local level. Additionally, our study served as an evaluation of a mental health intervention response for displaced persons. The Ministry sent a small group of mental health professionals to the displacement camps in order to lead group discussions. We found that among women in particular, any kind of support received, whether professional or from local supports (e.g., chiefs) was associated with reduced stress compared to women who reported that they were not able to get support (Zahlawi et al., 2019).

This study was also important because it provided a baseline for applying for funding for a follow-up study among the displaced population 2 years later. We received a grant from the National Geographic Society to explore the longer-term physical and mental health outcomes associated with the displacement in 2019. These data will provide important information about what strategies may buffer or amplify long-term health impacts of disasters like the one in Ambae. Finally, and perhaps most importantly, studies like this can provide a baseline of information to assist with developing and evaluating rapid interventions that target outcomes from the original needs assessment.

## 7. Lessons learned

In addition to the general value of the research conducted in the New Mexico and Vanuatu studies discussed in this article, the projects provide some important lessons for rapid research that we believe are helpful for planning similar types of studies. In this section, we reflect on our experiences in both projects, and how studies conducted on different types of disasters in different cultural contexts grant some generalizable lessons for future research in myriad settings.

### 7.1. Importance of local collaborators and locally experienced investigators

Research in both New Mexico and Vanuatu would not have been possible without existing relationships and the involvement of local collaborators and experts. In the New Mexico study, one member of the research team (Scott) has participated on health equity community boards for several years and has collaborated with the Doña Ana County Department of Health and Human Services and Doña Ana Wellness Institute on a number of health equity projects in the past. This existing relationship was important to be able to quickly establish a collaboration that would allow this project to move forward. In particular, having a community collaborator who works regularly with community health workers in the rural regions of the county was critical to successful recruitment efforts. While we recommend always including these community collaborators, during the COVID-19 pandemic, the research would have been impossible without this collaboration because usual routes of recruitment were closed (e.g., advertising in community centers, which were initially closed). Sharing the research ideas and plans early on with community collaborators, including the Doña Ana Wellness Institute has been critical to our ability to collect, analyze, and write up data in ways that are useful to our community partners.

Community collaborators and experts were similarly important in the Vanuatu research project. First, we had collaborators within the government who personally and professionally invested in what was happening on Ambae, which meant that we were able to get the necessary approvals relatively quickly. Second, we hired local research assistants who were well networked, meaning that they knew who to call or otherwise contact on the island in order to facilitate transportation, food, water, accommodation, and data collection. Relatedly, our local research assistants were comfortable working with people who they did not directly know and they were willing to forge new connections when necessary.

Both studies highlight the importance of a network of local community experts. We also want to state unequivocally that local collaborators are experts in their own communities and should be credited for their contributions via inclusion in authorship in all publications (which, at a minimum, can facilitate proposal writing for the collaborators for ongoing disaster relief aid), as well as providing timely and targeted reports back to local agencies in a position to use the findings.

We also note here the importance of locally experienced investigators; by this we mean inclusion of investigators (who may not be from the field site) who have previous experience conducting research within the affected community. We have already discussed author Scott's experience locally in New Mexico. In Vanuatu, our field team members had experience working in Vanuatu and on Ambae under “normal” circumstances, and thus were aware of how to interact with the communities and leverage local connections. Without the expertise of our research teams in both locations, our flexibility in responding to challenges as they arose would have been greatly reduced. Another advantage of having locally-experienced investigators is that it streamlines the process regarding applying for ethics approvals. In New Mexico, author Scott is a reviewer for the university IRB, and is highly familiar with their processes and procedures. In the case of Vanuatu, the principal investigator (author Dancause) was able to modify an earlier approved protocol, which otherwise may have delayed the project.

### 7.2. Large, cross-trained research team

Projects that implement multiple methodologies, that seek to sample a broad representation of the population, and that may require quick implementation of adjustment to research procedures require larger, cross-trained research teams. This means that any team member should be able to step into any part of the study procedures, and this limits introduction of error as well as potential disruptions to the study timeline.

In New Mexico, the project became more complex as we continued to adapt our research design due to changing COVID-19 restrictions in our state. It was helpful to have multiple team members who could work on specific parts of the project and replace each other as needed when individual circumstances changed. Additionally, our student members of the research team expanded our capacity to conduct our work. As we had prior mentoring relationships with the students, we were able to quickly train them to participate in all aspects of the project.

In Vanuatu, not all investigators were available to conduct fieldwork at the time of the displacement. The research team members were all trained at Binghamton University as graduate students in the Department of Anthropology, which enabled similar methodologies and processes to be implemented by whichever team members were able to conduct fieldwork. This meant that the investigators more involved in study design (Dancause and Olszowy) implicitly trusted the investigators more involved in fieldwork (Roome and Chan) to conduct the study as designed.

### 7.3. Access to rapid funding

Normal funding cycles for grants that may typically fund research on issues such as health disparities in marginalized communities were too extended to be feasible given the need to set up the research process and collect data quickly for both of these projects. Research funding is a challenge in any context, even under usual circumstances, but becomes increasingly difficult to find for rapid research. Federal sources in the US, like the NIH and NSF, have some mechanisms for rapid funding, but these tend to prioritize research in US states and territories, and funding is not guaranteed. Ideally, more institutions would provide researchers with specific funds that they can deploy in these kinds of circumstances, but that is not often a reality. During the course of the New Mexico research, the Department of Anthropology allowed authors Scott and Olszowy to repurpose their conference travel money for research. The research in Vanuatu would not have been possible without an undesignated funding line for research granted to author Dancause by her institution. We encourage other institutions to invest in this kind of funding to facilitate disaster or other types of rapid research.

Some organizations, such as the Natural Hazards Center, do exist specifically to fund rapid disaster research, and overall, funding a rapid project often takes a magpie-like approach to collecting small pots of funding from different sources, regardless of the context.

### 7.4. Adaptability and flexibility

An expectation that not if, but when things will go wrong is necessary for rapid disaster research. Researchers must maintain adaptability and flexibility (and we remind potential reviewers of rapid research manuscripts to remember this as well!). The New Mexico project could not follow the standard progression from design to data collection, analysis, and dissemination. Since the project results were critical for our partner organizations to access early on in the project, we moved among different aspects of the study more fluidly than we may have on other types of projects. We disseminated preliminary findings twice during the course of the study.

We also adjusted our research design to accommodate different recruitment strategies and larger than typical loss to follow-up. The mixed-methods approach contributed to our ability to adapt our project as circumstances changed. It also allowed flexibility for research participants to engage with the project with different levels of time commitment. While research processes are often less linear than they appear on paper, this project was particularly circular as we moved back and forth across the research process to make adjustments and provide regular reporting to our community partners.

In Vanuatu, authors Roome and Chan noted many instances where flexibility and adaptability were key in the field. Many of their experiences related to transportation difficulties on a tropical island without paved roads; the research team encountered several instances when they were unable to cross rivers, or where they were separated from some of their equipment, due to rainy weather. In these cases, they were able to utilize their knowledge of local networks to make changes to the survey schedule. It would not be practical in these situations to try to adhere to a strict schedule: researchers must be prepared to be flexible and to adjust their timeline accordingly.

### 7.5. Navigating infrastructural challenges

Existing infrastructure may enhance or impede the progress of research during “normal” times, and bureaucratic delays may become more apparent during rapid research, and particularly when the institution is also experiencing fallout from the disaster. In the case of our New Mexico project, the university funding structure was not able to respond as quickly as our funders. While our application for IRB approval was fast-tracked, approvals for expenditure of internal funds (i.e., repurposed travel awards) took longer. Part of the problems we encountered were due to changes in university operations related to various public health orders implemented by the State of New Mexico, but others were due to typical administrative checks and balances on funding sources. University systems need to be better prepared to shift to different approval processes that allow for faster approval when a researcher has funding for rapid research. Our funding from the Natural Hazards Center was awarded directly to the investigator, which made the process of using the funding much quicker (although record keeping was more onerous).

In Vanuatu, we received rapid ethical approval for our project and did not experience major delays in accessing funding due to university infrastructure, but had some difficulties in communicating with the Vanuatu Ministry of Health. Understandably, the Ministry was engaged in managing the disaster on Ambae, and availability of our usual contact with the Ministry (Director Tarivonda) was slower than usual. A wide network of existing contacts locally in this case was helpful in getting the project off the ground and approved by the local officials.

### 7.6. Survey resources for rapid researchers

Rapid research is unexpected, so if contemplating this kind of study at a different field site, awareness of what kinds of instruments are published and available is invaluable so that they can be quickly translated. We suggest the Natural Hazards Center website (https://hazards.colorado.edu/) as a good place to start looking for resources for survey (and general project) building for rapid research.

### 7.7. Avoiding undue burden for the local community

An overriding concern that we had during both projects was that we not become an additional burden on the local government and community as they were dealing with the disaster. In New Mexico, this included providing compensation for the survey and interview completion, in an amount appropriate for the time investment without being coercive among a population that is economically insecure (i.e., $10 grocery gift cards and $10 cash payments). In Vanuatu, the research team was particularly concerned about not using resources like transportation, food, and water that were directed at disaster relief. In both settings we also carefully considered the balance of value to burden in conducting this research for the community and local government, and made sure that preliminary results were made available to appropriate entities as soon as was feasible after the data were collected.

## 8. Conclusion and takeaways

In this article we have discussed challenges, opportunities, and lessons learned from two rapid disaster research studies among “hard-to-reach” communities. While there are many challenges to this type of research (e.g., lack of funding, difficult-to-access study populations, barriers to recruitment), research among these communities is important as well as practically and ethically feasible given appropriate planning. We highlight the following as major takeaways from this article given our literature review and experiences in the field. First, and perhaps most important, community/local collaboration and engagement is essential for all aspects of study design and implementation (e.g., planning, recruitment, analysis, and reporting). We argue that this is an important first step in mitigating potentially extractive practices that otherwise may alienate the study community from the research community. Second, researchers should pay attention to their own, and their team's, personal challenges and needs given the reality of researchers as human beings working under difficult conditions. Discussion of this point has grown in the literature during COVID-19, perhaps due to increased time for reflection borne of social isolation, but it is a positive direction for the field, particularly in terms of managing mental health and reducing burnout. Third, we found great value in mixed-method approaches to data collection, especially in terms of the project in New Mexico. Teams comprised of individuals with different academic strengths allowed for rapid deployment a study that used surveys, biomarkers, and REA, which would not have been possible without collaboration between authors Scott (a cultural medical anthropologist) and Olszowy (a biological anthropologist). Finally, we also highlight the need for adaptability/flexibility among the research team. No research product ever looks exactly like the initial research plan, and this is especially true for rapid research.

## Data availability statement

The original contributions presented in the study are included in the article/supplementary material, further inquiries can be directed to the corresponding author.

## Ethics statement

The studies involving human participants were reviewed and approved by New Mexico State University Institutional Review Board Institutional Committee on Ethics for Research Involving Humans, Université du Quebéc a Montréal Vanuatu Ministry of Health. The patients/participants provided their written informed consent to participate in this study.

## Author contributions

For DAC study, MS and KO contributed to conception and design of the study. HT, AM-L, EM, AG, and CM contributed to recruitment and data collection. MS, KO, HT, AM-L, and EM contributed to data analysis. For Vanuatu study, KO, KD, AR, CC, and LT contributed to study design. AR, CC, and BT conducted field work. LT and BT provided administrative support. All authors reviewed, had opportunity to revise, and approved the submitted manuscript.
